# Investigation of Integrated Reactive Multilayer Systems for Bonding in Microsystem Technology

**DOI:** 10.3390/mi12101272

**Published:** 2021-10-19

**Authors:** El-Mostafa Bourim, Il-Suk Kang, Hee Yeoun Kim

**Affiliations:** National NanoFab Center, Department of Nanostructure Technology, KAIST, 291 Daehak-ro, Yuseong-gu, Daejeon 34141, Korea; iskang@nnfc.re.kr

**Keywords:** multilayer reactive bonding, integrated nanostructure-multilayer reactive system, spontaneous self-ignition, self-propagating exothermic reaction, Pd/Al reactive multilayer system, Ni/Al reactive multilayer system, low-temperature MEMS packaging

## Abstract

For the integration of a reactive multilayer system (iRMS) with a high exothermic reaction enthalpy as a heat source on silicon wafers for low-temperature bonding in the 3D integration and packaging of microsystems, two main conflicting issues should be overcome: heat accumulation arising from the layer interface pre-intermixing, which causes spontaneous self-ignition during the deposition of the system layers, and conductive heat loss through the substrate, which leads to reaction propagation quenching. In this work, using electron beam evaporation, we investigated the growth of a high exothermic metallic Pd/Al reactive multilayer system (RMS) on different Si-wafer substrates with different thermal conduction, specifically a bare Si-wafer, a RuO_x_ or PdO_x_ layer buffering Si-wafer, and a SiO_2_-coated Si-wafer. With the exception of the bare silicon wafer, the RMS grown on all other coated wafers underwent systematic spontaneous self-ignition surging during the deposition process once it reached a thickness of around 1 μm. This issue was surmounted by investigating a solution based on tuning the output energy by stacking alternating sections of metallic reactive multilayer Pd/Al and Ni/Al systems that have a high and medium enthalpy of exothermic reactions, respectively. This heterostructure with a bilayer thickness of 100 nm was successfully grown on a SiO_2_-coated Si-wafer to a total thickness of 3 μm without any spontaneous upsurge of self-ignition; it could be electrically ignited at room temperature, enabling a self-sustained propagating exothermic reaction along the reactive patterned track without undergoing quenching. The results of this study will promote the growth of reactive multilayer systems by electron beam evaporation processing and their potential integration as local heat sources on Si-wafer substrates for bonding applications in microelectronics and microsystems technology.

## 1. Introduction

The adoption of integrated reactive multilayer systems (iRMS) as a bonding technique in microelectronics and micromechanical systems (MEMS) has recently started to gain more attention and traction in microsystems technology [1,2,3,4,5,6]. The reactive bonding at micrometer scales using bond frames with dimensions of a few micrometers makes reactive bond interface engineering using traditional freestanding reactive multilayer foils not practically feasible (i.e., due to the difficulty of foil handling, patterning, and positioning). Therefore, integration processing by the deposition/patterning or the patterning/deposition of reactive multilayer film systems directly on silicon wafers or other substrate components presents an interesting research challenge.

Reactive bonding uses a highly reactive nanoscale multilayer system as a self-heating source between joining substrates. The heat generation after an external initiation is created by a self-propagating exothermic reaction of the integrated RMS [4,5,7,8]. The integrated reactive bond induces local heat to the bonding interface; such limited heat/temperature quenches locally through the substrate material. This allows temperature-sensitive micro-devices located outside the interface and materials with different coefficients of thermal expansion (CTE) to be bonded without thermal damage. 

Generally, the reactive multilayer system typically consists of several alternating layers (up to hundreds) of two or more different reactant films combined as metal/metal, metal/oxide or metal/metalloid [9,10]. The bonding thermal energy source results from the exothermic reaction by the interdiffusion of adjacent material layers [8,11,12]. The required bonding energy level is chosen based on the negative enthalpy of the formation of material combinations [8,10]. It is also necessary to consider that the integrated reactive multilayer systems in bonding should be composed of highly reactive materials, which can release higher amounts of exothermic energy. This is important in order to assure a self-sustained propagating exothermic reaction and compensate for the heat absorbed in the bonding interfaces [2,8,13,14], such as the conductive heat losses through the hosting substrate or bonded component partners [14,15]. However, the use of highly reactive reactants has given rise to different issues. The most disadvantageous is the spontaneous self-ignition of RMS during thin reactant layer deposition. With existing standard sputtering equipment without an active substrate cooling system, it is not possible to deposit any number of individual layers; after just a few layer superpositions, these piled layers self-ignite and react before the coating process has reached the final requested total layer number [2,8].

Other issues include the reaction initiation effect during RMS handling, premature intermixing at the interfaces of stacked RMS reactant layers [12,16,17,18], and RMS ignition in a strong explosive exothermic reaction leading to the vaporization or ejection of the reaction product [19,20,21,22]. It should also be noted that the partly self-reacted RMS and pre-intermixed reactant layer interfaces are considered to be among the main factors preventing the reliable initiation of self-propagating exothermic reactions at room temperature in integrated RMSs [12,16,17,18]. These partially consumed reactants reportedly reduce the potential heat energy required for the reaction ignition and propagation [12,16,17,18].

The goal of this investigation is to integrate highly reactive reactant film layers directly on Si-wafer substrates using a conventional electron beam deposition system that is not equipped with an active substrate cooling setup. To avoid the delamination of deposited layers and reduce internal mechanical stresses within the reactive multilayer system, the total thickness of the integrated reactive multilayer system (iRMS) should not exceed 5 μm [2,7,8]. Furthermore, to overcome the limitation of the standard photoresist lift-off patterning technique used in the preparation of iRMS pattern samples, the maximum total thickness of the deposited RMS was limited to 3 μm. Hence, a smaller iRMS thickness contributes to cost-efficiency by using smaller amounts of noble metals, which are commonly used as reactants in RMSs that supply high thermal energy.

In this work we investigated the integration of Pd/Al RMS on a silicon substrate coated with different thermal barrier layers. The RMS deposition was performed on a bare silicon wafer, a RuO_x_-buffered Si-wafer, a PdO_x_-buffered Si-wafer, and a SiO_2_-coated Si-wafer. It has been shown that the Pd/Al iRMS can, in principle, be used for reactive bonding. However, as Pd/Al iRMS is highly exothermically reactive, spontaneous self-ignition and propagating reactions during deposition could happen frequently when the thickness of the deposited RMS reaches a critical value. It was also observed that for a substrate that has a low thermal conductivity, the high-confined released heat and its associated high temperature in the ignited iRMS led to reaction propagation in an explosive-like manner with a partial evaporation/ejection of the reaction product. To overcome these issues, we attempted to mitigate the reactants’ reactivity effect by alternately stacking a highly exothermic heat-releasing RMS with a relatively lower or medium RMS. The alternating combination of multi-sections of a pure metallic stack Pd/Al RMS with a high enthalpy of mixing with a pure metallic stack Ni/Al RMS that had a moderate enthalpy of mixing, deposited together on a SiO_2_-coated Si-wafer, demonstrated the successful growth of a full, intact iRMS with a thickness of 3 μm with no spontaneous self-ignition surging. It was also confirmed by thermal measurements that this combination, by alternately stacking different RMSs with different exothermic heat enthalpy, is an efficient way of modulating the reaction heat release. Moreover, these grown multi-section Pd/Al-Ni/Al iRMS samples exhibited ignition, which led to a self-sustained propagating reaction that was feasible by a simple triggering with an electrical DC pulse at room temperature.

## 2. Materials and Experimental Techniques

The reactive multilayer thin-film systems were deposited using an electron-beam evaporation system (KVE & T-C500200, Korea Vacuum Tech, Ltd., Gimpo-si, Korea). The substrate holder of this system was not equipped with an active substrate cooling accessory. The substrates were prepared in accordance with the standard RCA cleaning method. Depositions were performed at room temperature and at a base pressure of 5 × 10^−8^ Torr. Generally, such deposition conditions promote nano-grain formation in a columnar-like structure that is basically controlled by the reduced atomic mobility of the deposited species on the substrate, and self-shadowing during film growth [23,24]. The substrates on which the iRMS was deposited were 4” (100) bare Si-wafers, Si-wafers coated with thermally grown SiO_2_ (1 μm thick), and Si-wafers buffered either with SiN_x_/RuO_x_/Cr or SiN_x_/PdO_x_/Cr stacks with respective layer thicknesses of 50 nm/60 nm/10 nm. The iRMS systems were deposited by an alternating e-beam evaporation of the reactant layers (Pd and Al for Pd/Al RMS and Ni and Al for Ni/Al RMS), from high purity targets (Al 99.9995%, Pd 99.9995%, and nickel 99.9995%). For the buffering (Ru, Pd) and adhesive (Ti, Cr) layers with respective thicknesses of 60 and 10 nm, the deposition was from targets with respective specific purities of 99.95%, 99.9995% and 99.9995%, 99.9995%. The thermal oxidation of Ru- and Pd-buffering Cr/Si-wafers was performed in a quartz tube furnace with flowing oxygen at 350 °C for 30 min (MTI Korea). After oxidation, the elemental distribution in the thickness direction was achieved by Auger electron spectroscopy (AES) using the depth profile technique (VG Scientific MicroLab 350). The SiN_x_ diffusion barrier layer, capping RuO_x_ and PdO_x_-buffered Si-wafers, was grown by a low-pressure chemical vapor deposition method at 200 °C (LPCVD; E-1200, Centrotherm).

The thickness ratio of the bilayer reactants was determined in relation to a stoichiometric ratio of 1:1, corresponding to the maximum heat release from both Pd/Al and Ni/Al RMS. The Pd/Al-iRMS is a high-energy system and the Ni/Al-iRMS is a medium-energy system. The standard formation enthalpies for the stoichiometric ratio of 1:1 used here in this work for both systems are, respectively, −90 and −59 kJ/mol [25,26]. The different investigated iRMSs were grown with bilayer periods (δ) of either 50 nm, 100 nm or 200 nm, and total heights of either 1 μm, 2 μm, 2.4 μm or 3 μm.

**Figure 1:**
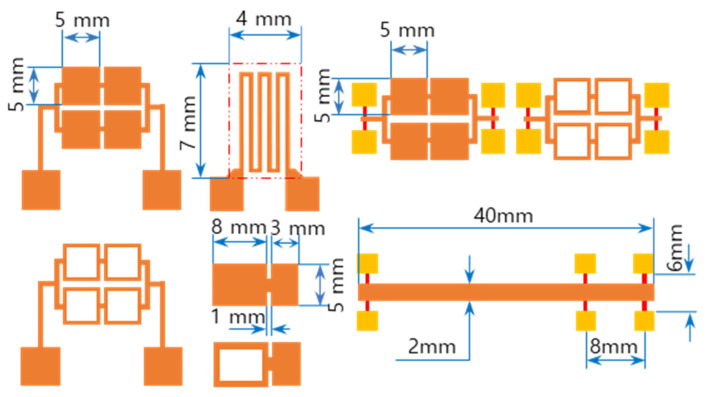
Different integrated reactive multilayer systems (iRMS) pattern shapes used for the observation of reaction propagation behavior (the initiation of the iRMS ignition using a Joule heating effect can be carried out by the application of the DC bias current either directly on the reactive pads or through gold contacts (yellow pads) connected with a titanium line (red stripes)).

The RMS films were integrated by a lift-off technique. The different tailored RMS host geometries on the photoresist-coated Si-wafer were a photo-lithographically transferred replica of motifs patterned on a chromium glass photomask using a contact aligner (EVG 640, EV Group, Austria) for ultraviolet light exposure up to 170 mJ/cm^2^. The patterned geometry of iRMS traces emerged by dissolving the photoresist with acetone and, thus, systematically releasing the metal deposited on it. All iRMS samples were patterned mainly in shapes of small, squared pads connected either to rectangular frames or to serpentine paths, simple long stripes, and some simple large rectangular areas (see Figure 1). Such patterning would be practical for measuring, under a high-speed recording camera, the front speed of the propagating reaction as well as confirming the reaction’s ability to propagate through different complicated bonding paths. The square pad in the iRMS patterns served as a starting local area for the ignition triggering.

Microstructure, composition, and elemental distribution analyses of the as-deposited iRMS films and the reaction products were performed on the lamellae of selected cross sections by using a field emission transmission electron microscope (FE-TEM, JEM-2100F HR, JEOL, Japan) and a Cs-corrected scanning transmission electron microscope (STEM, JEM-ARM200F, JEOL, Japan) equipped with an energy dispersive X-ray spectrometer (EDS) for elemental mapping. Transmission electron microscope (TEM) image scans were taken with an emissive gun operating at an acceleration voltage of 200 kV, and EDS analyses were performed with an acceleration voltage of 15 kV and a step size of ~0.25 μm. The lamellae-like samples were prepared by means of a dual-beam focused ion beam apparatus (FIB, Helios NanoLab, FEI, Netherlands) equipped with an omniprobe lift-out system.

The reaction propagation speed was determined by recording the reaction propagation path in the patterned iRMS with a high-speed camera (i-SPEED 221, iX Cameras, Frames at Max. Res.: 600 FPS, European Union). The initiation of the self-propagating reaction front was made by setting a DC power supply to 10 V/max. 1 A, which was applied between two tungsten needle probes with sharp tips.

The reaction heat of the as-prepared reactive multilayer systems was assessed by using a differential scanning calorimeter (DSC) (NETZSCH, DSC 404F1, Germany). The samples were freestanding, reactive multilayer nano-foil strips previously integrated by depositing an RMS film on 250 nm sacrificial Cu-layer-coated Si-wafers. The nano-foil strips were released from wafers by selective Cu-layer etching in an acid immersion solution of ammonium persulfate (20% (NH_4_)_2_S_2_O_8_ + H_2_O). DSC measurements were carried out on samples of ~5 mg placed in an alumina crucible and heated in a temperature ranging from room temperature (RT) to 800 °C at a constant rate of 40 °C/min in an atmosphere of N_2_ and Ar gases flowing at rates of 50 and 20 mL/min, respectively.

In cases where the cross section preparation of intact TEM specimens by FIB cutting was not possible, the morphological surface characterization of iRMS was carried out using a digital optical microscope (KEYENCE VHX-6000) and the crystalline structure and phase composition were examined by an X-ray diffractometer (XRD, SmartLab, Rigaku Corporation, Japan) operated in Bragg-Brentano θ-2θ geometry mode with a CuKα radiation source (k = 1.5405 Å) at 40 kV and 40 mA. The scans were performed with a 2θ step size of 0.02° in the 2θ range from 20° to 90°.

## 3. Results and Discussion

Before presenting the results of this work, we need to give information about our targeted experiments that allowed us to obtain the following experimental results. Our calculation based on thermal transfers (not presented here) showed that in order to obtain ignition at room temperature with a sustained propagating reaction in Pd/Al iRMS with a bilayer period of 100 nm grown directly on a Si-wafer, a reactive multilayer stack with a total thickness over 5 μm is needed. However, this is technically contradictory to what is mentioned in the introduction (the limitations of mechanical stress and photoresist patterning). Furthermore, the calculated thickness leading to ignition with reaction propagation at room temperature for a Pd/Al RMS with a bilayer period of 100 nm grown on a SiO_2_-coated Si-wafer was determined to be around 1.6 μm and higher. However, the high thermal insulating SiO_2_ layer did not help to reach the aimed iRMS thickness due to heat accumulation, which led to spontaneous self-ignition and a propagating reaction during deposition. Therefore, to remedy such issues by assuring heat sinking during multilayer stack deposition and heat damming during the self-sustained propagating reaction, a technical solution based on building an instant thermal barrier was carried out by inserting thin metal oxide buffer layers of either RuO_x_ or PdO_x_ between the Si-wafer and the iRMS film. These buffering layers, with a thermal conductivity that was much higher than that of SiO_2_ during RMS deposition (for comparison, the thermal conductivity value of wafer-covering layers and the equivalent thermal conductivity of their superposition are given in Table 1), would easily dissipate the accumulated heat in the multilayer stack down to the Si-substrate sink and would, thereby, avoid self-ignition. In contrast, in an effective RMS ignition test, for which a sustained propagating reaction is expected, the high temperature attained as well as the dissipated heat would diffuse oxygen down from the oxide buffer layer and simultaneously oxidize the Si-wafer surface progressively along the propagation path. This results in the formation of an instant local thermal barrier interface, thereby avoiding self-propagating reaction quenching.

Further, as supplementary information, Pd/Al iRMS grown on bare Si-wafers, which theoretically requires a thickness greater than 5 μm to be ignitable at RT, as well as on SiO_2_-coated Si-wafers, which practically undergoes a systematic spontaneous self-ignition during the deposition process, was prepared and investigated for comparison.

### 3.1. Pd/Al iRMS Grown on a Bare Si-Wafer and a RuO_x_-Buffered Si-Wafer (iRMS Total Thickness ~ 1 μm)

#### 3.1.1. Characterization of the as-Deposited Pd/Al-iRMS

**Figure 2:**
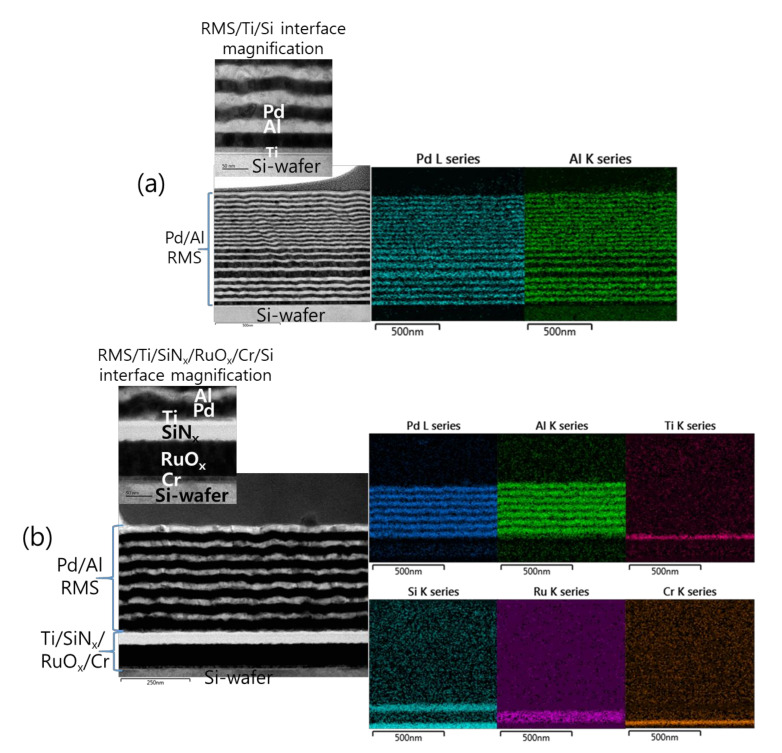
Transmission electron microscope (TEM) cross sections (**left**) and corresponding energy dispersive X-ray spectrometer (EDS) elemental mappings (**right**) of as-deposited Pd/Al RMS on the Ti/Si-wafer (**a**), and on the Ti/SiN_x_/RuO_x_/Cr/Si-wafer (**b**).

Figure 2 shows the microstructural analyses by TEM and the corresponding EDS elemental mappings of a Pd/Al-iRMS consisting of 20 bilayers with a period of 50 nm and a stoichiometric ratio of Pd:Al = 1:1. The iRMS was patterned and deposited simultaneously on two different substrate surfaces: a bare Si-wafer (Figure 2a) and a RuO_x_-buffered Si-wafer (Figure 2b). For the TEM cross-sectional analysis of the Pd/Al-iRMS deposited directly on the bare Si-wafer, a successful multilayer stack having a layered microstructure with a sharp separation of single layers was observed. The EDS analysis, in turn, confirmed the presence of alternating metallic layers of Pd and Al (Figure 2a). However, for the Pd/Al-iRMS deposited on the RuO_x_-buffered Si-wafer, the TEM cross section demonstrates that a spontaneous self-ignition occurred and was instantly accompanied by an explosive detachment of the reacted product. Thus, Figure 2b shows the layers deposited immediately afterwards when the previous deposited layers had been reacted and detached by self-ignition. The EDS analysis, in turn, confirmed the stability of the used buffering Ti/SiN_x_/RuO_x_/Cr stack layers and the remaining alternately deposited metallic layers of Pd and Al (Figure 2b).

#### 3.1.2. Characterization of the Reacted Pd/Al-iRMS 

**Figure 3:**
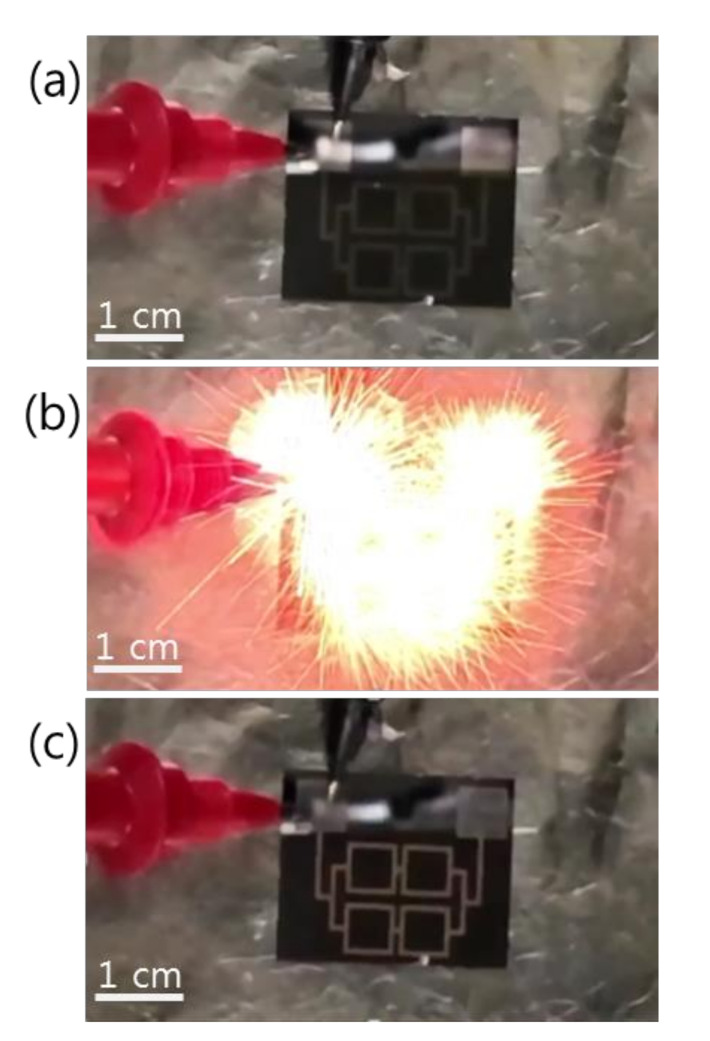
Reaction propagation across a patterned Pd/Al-iRMS of squared frames grown on a Si-wafer. (**a**) Initiation step; (**b**) Maintained propagating reaction step; (**c**) Reaction product morphology after the reaction step.

Figure 3 shows the reaction propagation across a patterned Pd/Al-iRMS of square frames grown on a Si-wafer. Since the total multilayer stack thickness (~1 μm) was lower than the minimal value (~5 μm) calculated for a self-maintained propagating reaction, preheating of the substrate at 100 °C for 5 min was indeed needed for the Pd/Al-iRMS ignition. The three snapshots presented in Figure 3, respectively, illustrate the ignition step initiated by a DC pulse of 10 V/max. 1 A (Figure 3a), the reaction propagation step manifested in an explosive yellow bright glow (Figure 3b), and the final morphology of the reaction product after the propagating reaction occurred (Figure 3c). The same explosive reaction propagation behavior was observed for the Pd/Al-iRMS grown on the RuO_x_-buffered Si-wafer; however, higher preheating at 150 °C for 5 min was needed to initiate the reaction propagation, since the successfully deposited iRMS thickness was smaller.

**Figure 4:**
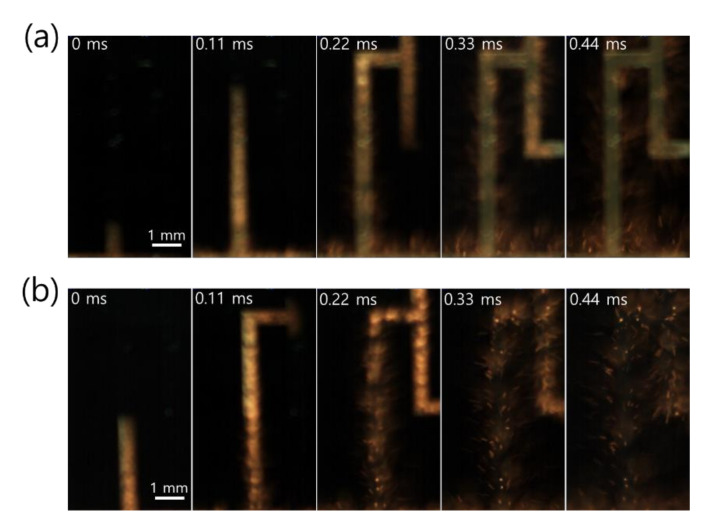
High-speed snapshots of the explosive reaction propagation in Pd/Al-iRMS, from left to right. (**a**) Pd/Al-iRMS grown on a bare Si-wafer; (**b**) Pd/Al-iRMS grown on a RuO_x_-buffered Si-wafer.

Close snapshots taken with a high-speed camera, used to determine the front speed of the propagating reaction, showed that the reaction behavior of Pd/Al-iRMS on the Si-wafer manifested an explosive combustion process with small quantities of ejected product particles (Figure 4a). Meanwhile, for the Pd/Al-iRMS on the RuO_x_-buffered Si-wafer, a fiercer explosive combustion process with larger quantities of ejected product particles, leading to an almost full detachment of the patterned structure, was observed (Figure 4b). The propagation speeds of the reaction front, determined by the reactions in the Pd/Al-iRMS grown on the bare Si-wafer and the RuO_x_-buffered wafer, were 38 and 50 m/s, respectively.

The explosive reaction process with a high ejection or evaporation of the reaction product could be related to the attainment of a high local temperature. In fact, the small bilayer period of 50 nm that generates fast reaction propagation leads to a very high temperature level, as the produced heat does not have enough time to dissipate through the substrate. It should also be mentioned that a Pd/Al-iRMS with a smaller bilayer period has a higher self-initiation risk than systems with a larger bilayer period. Therefore, to overcome the bilayer size issues, only the iRMS with a bilayer period higher than 50 nm was investigated in the following study. This approach, according to the literature, should be seen as beneficial since reactive multilayer systems with bilayer periods between 100 nm and 200 nm are almost free of residual stress. RuO_x_ buffering is also suspected to provide an additional heat supply. In fact, RuO_x_, at a high ambient temperature, could undergo more exothermic oxidation transformations such as RuO_3_, RuO_4_ etc. [32,33,34], which highly increase the temperature in the ignited iRMS and, thus, lead to the evaporation and ejection of the reaction product. Hence, in the subsequent experiments, Si-wafer buffering is prepared by replacing the RuO_x_ with a thin PdO_x_ buffer layer. This one possesses a high chemical stability in high temperatures [34,35].

### 3.2. Pd/Al-iRMS Grown on SiO_2_-Coated and PdO_x_-Buffered Si-Wafers (iRMS Total Thickness ~2 to 2.4 μm)

#### 3.2.1. Microstructural Characterization of the as-Deposited Pd/Al-iRMS

**Figure 5:**
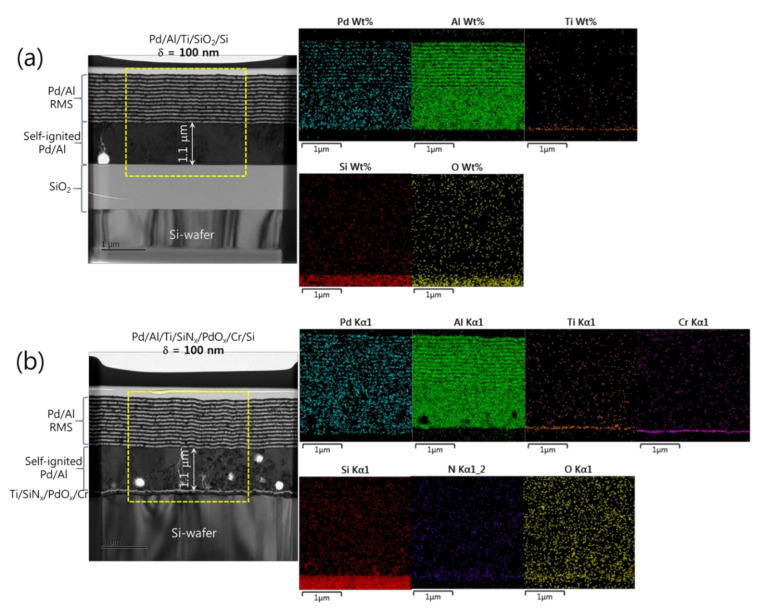
TEM cross sections (**left**) and corresponding EDS elemental mappings (**right**) of Pd/Al-iRMS with a bilayer thickness of δ = 100 nm and a bilayer number of N = 24. (**a**) Pd/Al-iRMS grown on a SiO_2_-coated Si-wafer; (**b**) Pd/Al-iRMS grown on a PdO_x_-buffered Si-wafer.

Figure 5a,b show microstructural analyses by TEM and corresponding EDS elemental mappings of Pd/Al-iRMS structures grown on SiO_2_-coated and PdO_x_-buffered Si-wafers with a multilayer structure design composed of N = 24 bilayers with δ = 100 nm and N = 12 bilayers with δ = 200 nm, respectively. For each bilayer period, the iRMS frames were patterned and deposited simultaneously on both SiO_2_-coated Si-wafer and PdO_x_-buffered Si-wafer. In Figure 5a,b for the Pd/Al-iRMS with δ = 100 nm, it can be seen that spontaneous self-ignition occurred simultaneously on both the prepared Si-wafer-types, after about a 1.1 μm thickness of multilayer stack deposition corresponding to around 11 deposited bilayers. The intact reactive multilayers, grown next on the intermixed part, show for both prepared Si-wafer-types a neat, layered microstructure with a sharp separation between single layers with no significant premature intermixing. The thicknesses of the deposited reactant layers approached the expected nominal values corresponding to a 1:1 atomic ratio. EDS analyses also, by a chemical composition probing of the grown microstructure, confirmed the presence of an Al_x_Pd_y_ reaction product, which resulted from spontaneous self-ignition, and an additional stack on it of alternating reactant layers with Al and Pd compositions (Figure 5a,b). 

**Figure 6:**
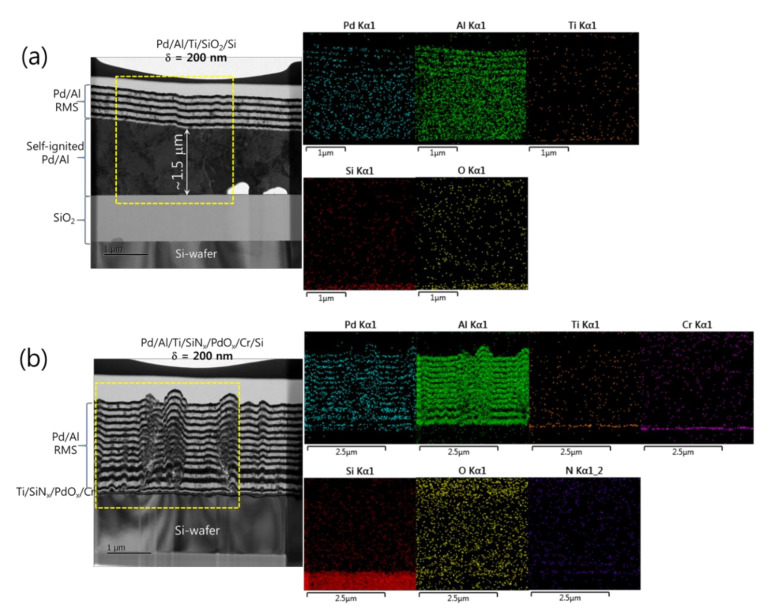
TEM cross sections (**left**) and corresponding EDS elemental mappings (**right**) of Pd/Al-iRMS with a bilayer thickness of δ = 200 nm and a bilayer number of N = 12. (**a**) Pd/Al-iRMS grown on a SiO_2_-coated Si-wafer; (**b**) Pd/Al-iRMS grown on a PdOx-buffered Si-wafer.

In Figure 6 for the Pd/Al-iRMS with δ = 200 nm and a total bilayer number of N = 12, different results were obtained. It can be seen that the spontaneous self-ignition and the resulting intermixing product occurred only for the Pd/Al-iRMS grown on the SiO_2_-coated Si-wafer (Figure 6a), whereas the Pd/Al-iRMS grown on the PdO_x_-buffered Si-wafer showed continuous stacking of individual (Al, Pd) reactant layers, confirming the absence of any self-ignition surging (Figure 6b). The stacking faults observed in the Pd/Al reactant layers are likely linked to the rough morphology of the surface of the PdO_x_ buffer layer. The reacted product on the SiO_2_-coated Si-wafer formed when the Pd/Al reactants layer stack reached a thickness of about 1.5 μm, after which around five Pd/Al bilayers were further deposited. The delay of the spontaneous self-ignition in this iRMS with a 200 nm Pd/Al bilayer grown on a SiO_2_-coated Si-wafer, compared to a 100 nm Pd/Al bilayer grown on either SiO_2_ or PdO_x_ layers covering Si-wafers, could be due to fewer layer interfaces (potential sites of premature intermixing), which resulted in less barrier hindrance to heat conduction and, hence, less heat accumulation in the iRMS, thereby avoiding precocious start of spontaneous ignition. Again, EDS analyses of the Pd/Al-iRMS with a 200 nm bilayer period confirmed, for the iRMS on a SiO_2_-coated Si-wafer, the presence of the intermetallic Al_x_Pd_y_ reaction product composition with an additional deposited Pd/Al layered structure over it (Figure 6a). On the other hand, for the iRMS grown on the PdO_x_-buffered Si-wafer, the analysis probed a full layered structure of a continuous alternating reactant stack of Pd and Al compositions (Figure 6b).

#### 3.2.2. Microstructural Characterization of the Reacted Pd/Al-iRMS 

**Figure 7:**
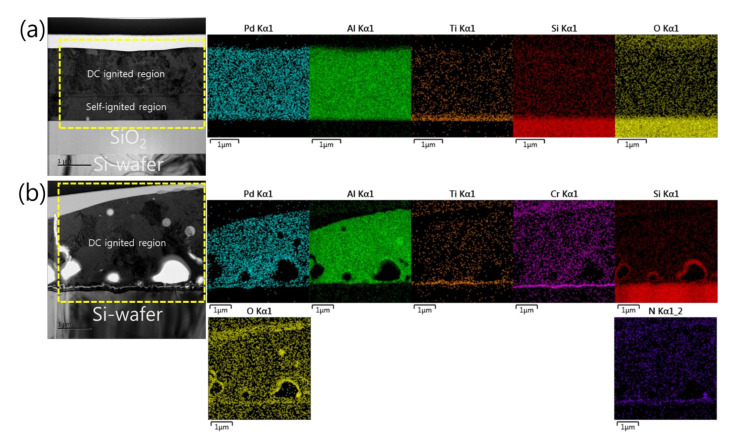
TEM cross sections (**left**) and corresponding EDS elemental mappings (**right**) of the Pd/Al-iRMS reaction product microstructure after the exothermic propagation reaction. (**a**) Pd/Al-iRMS with a bilayer thickness of δ = 100 nm grown on a SiO_2_-coated Si-wafer; (**b**) Pd/Al-iRMS with a bilayer thickness of δ = 200 nm grown on a PdO_x_-buffered Si-wafer.

The following presents the microstructural analyses of the reaction product produced by electrically igniting the Pd/Al iRMS. The self-propagating exothermic reaction was observed only after preheating each of the four investigated samples. For samples that underwent spontaneous self-ignition during deposition (i.e., the Pd/Al-iRMS grown on SiO_2_-coated Si-wafers with δ = 100 nm and 200 nm; and the Pd/Al-iRMS grown on a PdO_x_-buffered Si-wafer with δ = 100 nm), ignition triggering happened at a preheating temperature of ~150 °C for 10 min. Meanwhile, for the sample with entire layer stack deposition (Pd/Al-iRMS grown on a PdO_x_-buffering Si-wafer with δ = 200 nm), ignition triggering happened at a preheating temperature of ~100 °C for 10 min. For the convenience of presentation, Figure 7 depicts the microstructure of the reaction product of the samples of Pd/Al-iRMS with δ = 100 nm grown on a SiO_2_-coated Si-wafer and Pd/Al-iRMS with δ = 200 nm grown on a PdO_x_-buffered Si-wafer, after the self-propagating exothermic reaction passed through the reactive Pd/Al multilayer system. The individual Al and Pd layers are completely intermixed and, thus, no layered structure can be seen (TEM images, Figure 7a,b). EDS analyses also clearly confirm that all the Al and Pd reactant elements are completely intermixed, and no individual layer can be detected (EDS mapping, Figure 7a,b). Hence, since the ignition of the Pd/Al multilayer stack system had a stoichiometric ratio of Pd:Al = 1:1, it can systematically be demonstrated that the formed reaction product phase consisted of a homogeneous AlPd intermetallic compound.

Note that the difference in temperatures of the preheating process, which is required to ignite and maintain the self-propagating reaction process, indicates that in the iRMS samples that underwent spontaneous self-ignition during multilayer deposition, the preformed AlPd intermetallic reaction product located under the additional upper Pd/Al-iRMS stack could sink more heat into the Si-wafer substrate. Thus, for the upper iRMS to be ignited, it required more energy and a higher temperature.

**Figure 8:**
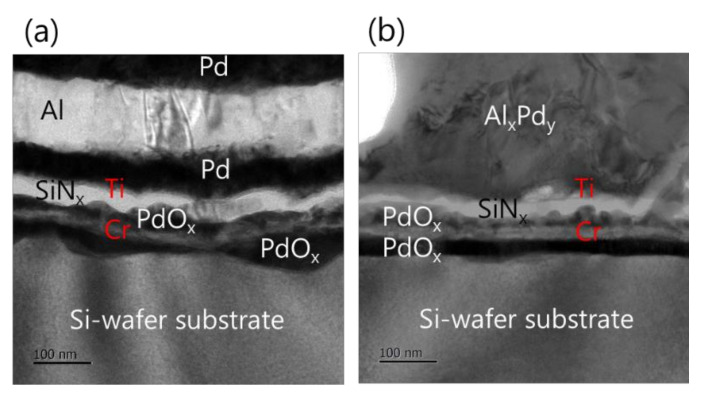
TEM images showing the interface structure configuration of the iRMS grown on a PdO_x_-buffered Si-wafer. (**a**) As-deposited RMS; (**b**) After reaction ignition.

Furthermore, the analysis of the interface structure between the iRMS and Si-wafer substrate showed that no remarkable change occurred before and after the ignition of the samples. For the Pd/Al-iRMS grown on the SiO_2_-coated Si-wafer substrate, the interface structure configuration was found to be similar before and after the reaction propagation. For the Pd/Al-iRMS grown on the PdO_x_-buffered Si-wafer substrate, the iRMS/(Ti/SiN_x_/PdO_x_/Cr)/Si-wafer interface of the as-deposited, and then ignited, iRMS is presented by the cross-sectional TEM images in Figure 8a,b. It is shown that, as a part of the PdO_x_ buffer layer diffused down through the adhesive Cr layer, the result was that the adhesive Cr layer was totally pushed a few nanometers up and, thus, the PdO_x_ established direct contact with the Si-wafer substrate. Note that the interface structure configuration was found to be practically similar for both situations, before and after the reaction ignition. 

**Figure 9:**
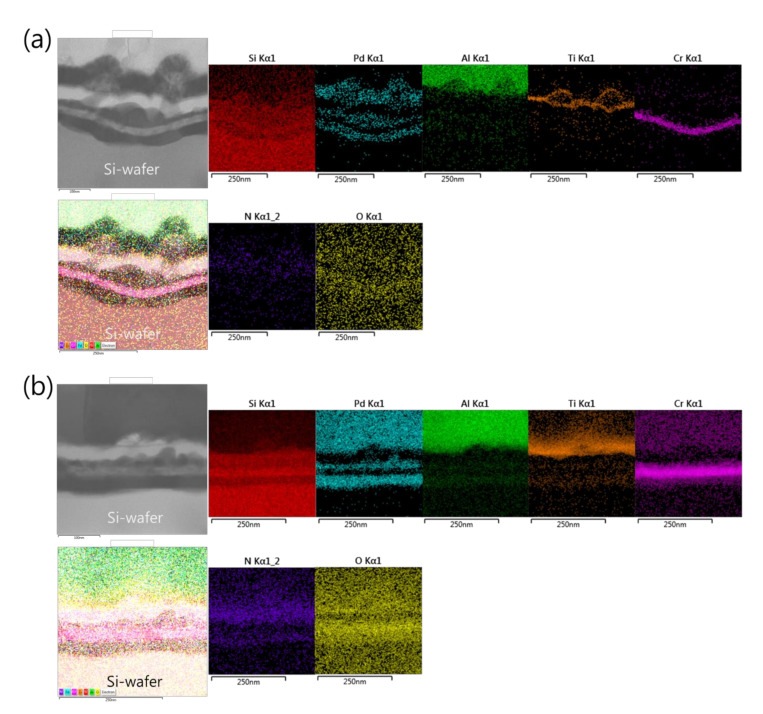
Scanning transmission electron microscope (STEM) images (upper left) and corresponding EDS elemental mappings of the interface structure configuration of the iRMS grown on a PdO_x_-buffered Si-wafer. (**a**) As-deposited RMS; (**b**) After reaction ignition.

**Figure 10:**
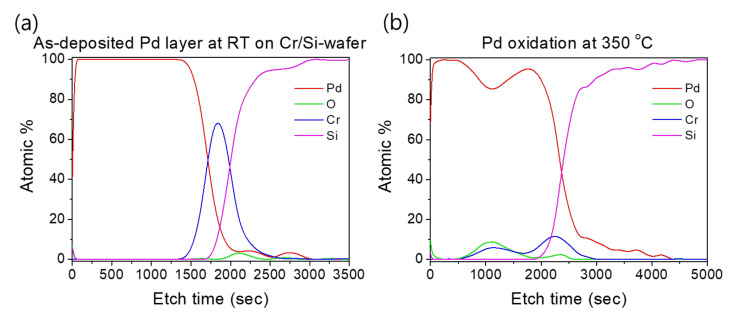
AES profiles showing the effect of temperature on Pd and Cr distribution during the thermal oxidation process. (**a**) As-deposited Pd film on a Cr-coated Si-wafer; (**b**) Pd film oxidation at 350 °C for 30 min.

Such an interface structure configuration was also confirmed by STEM and EDS analyses, as shown in Figure 9. In fact, Pd diffusion happened, in the preparation of the buffered Si-wafer, during the 60 nm thick Pd film thermal oxidation. This behavior was elucidated by an AES analysis of the as-deposited, room temperature Pd film on a Cr-coated Si-wafer substrate and its thermal oxidation at 350 °C for 30 min (Figure 10). For the as-deposited Pd film at room temperature, a neat separation between Pd and Cr layers and the Si-wafer was probed (Figure 10a). However, for the thermally oxidized Pd film, the deep diffusion of the Pd element through the Cr layer and inward into the Si-wafer with oxidation limited at the Cr layer was probed (Figure 10b). It is also worth mentioning that the operation of the instantaneous oxidation of the Si-substrate surface during the iRMS ignition remains effective. However, given that the amount of oxygen stored in the PdO_x_ buffer layer was not very high, the diffusion and distribution of the oxygen into the Si-substrate surface could not be detected clearly by the probing EDS system used in this study.

#### 3.2.3. Thermal Characterization of Pd/Al-iRMS 

**Figure 11:**
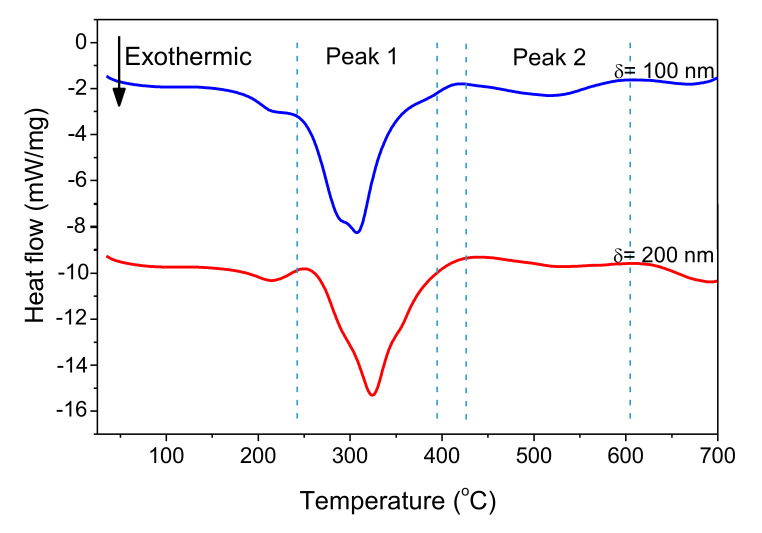
DSC curves of Pd/Al-iRMS with different bilayer thickness.

Figure 11 shows DSC curves of freestanding nano-foils of Pd/Al-RMS with bilayer thicknesses of 100 and 200 nm. These RMS nano-foils were released from the SiO_2_/Si-wafer coated with a Cu layer of 250 nm by immersion in an acidic solution bath. Irrespective of the bilayer period, two larger exothermic peaks were produced with their start and end temperatures having nearly the same values. The first peak began at around 240 °C and the second peak started at around 425 °C. Furthermore, it can be seen that the first peak height increased with increasing bilayer period thickness, a behavior commonly related to the stored energy loss by the higher intermixed interface volume in the reactive system having a lower bilayer period. The heat released was calculated using the principle of integrating the heat flow with respect to time. The total heat of the reaction was determined to be 797.3 J/g for the system with a 100 nm bilayer and 778.6 J/g for the system with a 200 nm bilayer. These detected reaction enthalpy values are significantly lower than the expected theoretical value of 1260 J/g [8,36]. Therefore, the relatively large difference between the experimentally measured reaction heat and the theoretically predicted value could be mainly attributed to the formation of the reaction product by the spontaneous self-reaction that happened in the prior deposited layers by self-ignition into the e-beam evaporator chamber during the deposition process.

**Figure 12:**
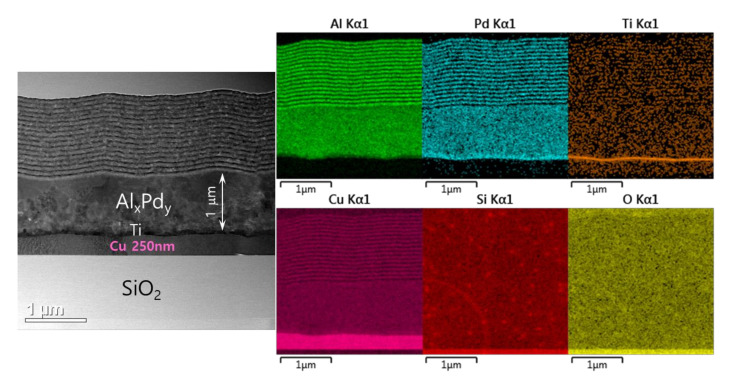
TEM cross section microstructure (**left**) and corresponding EDS elemental mappings (**right**) of Pd/Al-iRMS with a bilayer thickness of δ = 100 nm grown on a 250 nm Cu film-coated SiO_2_/Si-wafer.

The spontaneous self-ignition occurred in the prepared Pd/Al-RMS freestanding nano-foils despite the intercalation of a highly thermal-conductive layer of Cu material (which can sink the accumulated heat in the reactant layers and, thus, avoid the reaction ignition), was confirmed by the observed formation of the reaction product Al_x_Pd_y_ in the microstructural characterization depicted in Figure 12. This figure presents the TEM microstructure and the corresponding EDS analysis obtained from a Pd/Al-iRMS with a bilayer period of 100 nm and an expected total thickness of 30 bilayers grown on a SiO_2_/Si-wafer coated with a thin Cu layer of 250 nm. The cross-sectional images clearly depict the presence of three surface sections, respectively, corresponding to the lower copper layer, the reaction product formed by spontaneous self-ignition, and the deposited intact Pd/Al iRMS layers at the top. The thickness of the Al_x_Pd_y_ reaction product stated that the self-ignition was initiated after around 1 μm thickness of the earlier deposited Pd/Al stack layers. This scenario was also similar to the spontaneous self-ignition that had taken place, at around 1 μm of layer stack deposition, in the Pd/Al-iRMS with a bilayer period of 100 and 200 nm grown directly on a SiO_2_-coated Si-wafer.

From the deposition experiments described above, the investigated Pd/Al-iRMS with a bilayer period of 100 nm showed spontaneous self-ignition accompanied systematically by a sustained exothermic propagation reaction for all stacked Pd/Al reactive multilayers with thicknesses exceeding 1 μm, deposited either directly on a SiO_2_/Si-wafer (iRMS on a high-thermal insulating substrate), metal/SiO_2_/Si-wafer or metal oxide/Si-wafer (iRMS on a moderate thermal insulating substrate). Such behavior can apparently be related to the Pd/Al system’s high exothermic enthalpy of formation (ΔH_Al-Pd_ ≈ 90 kJ/mol-atom), a value that is substantially higher than the 30 kJ/mol-atom required for generating self-sustained propagating reactions in thin reactive multilayer films [10]. Accordingly, small nanometric inter-diffusions at the Pd/Al reactants interfaces, even for a system with a small number of bilayers, could release enough energy output for spontaneous self-ignition triggering and to simultaneously sustain continuous exothermic propagation reactions on the silicon substrate. Therefore, the main issue to be addressed regarding the Pd/Al-iRMS is the low excitation energies causing instabilities, which lead to an enhanced risk of spontaneous reaction ignition as well as pre-intermixing activation at the reactant interfaces during the deposition processes.

**Figure 13:**
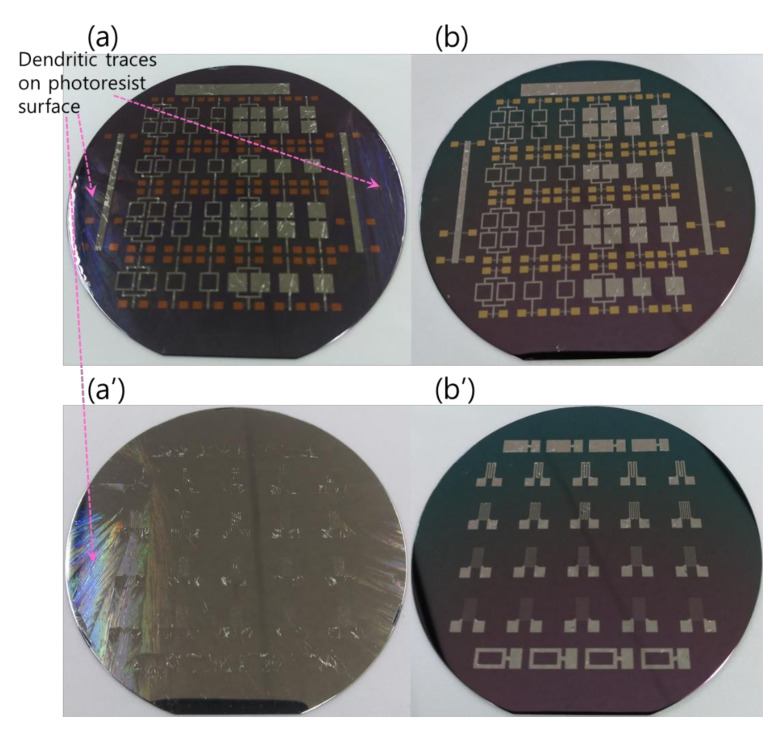
Dendritic traces on a photoresist film surface after spontaneous self-ignition. (**a**,**a’**) Wafer surfaces before photoresist lift-off; (**b**,**b’**) Wafer surfaces after photoresist lift-off.

Since, in this work, the photoresist was adopted as the material for the lift-off patterning of the integrated RMS structures, further information worth noting is related to the interaction of the RMS and the photoresist. Because of the very low thermal conductivity of the photoresist, the few bilayer number depositions of the reactive Pd/Al system directly on the photoresist would undergo spontaneous self-ignition and a continuous propagation reaction spreading over the whole four-inch Si-wafer surface. In fact, the high reactive Pd/Al system, the mixing enthalpy and the low thermal conduction through the photoresist, caused the spontaneous reaction to occur in a strongly explosive manner, simultaneously accompanied by the detachment/ejection of the reacted product, while leaving the photoresist film still tightly adhered to the wafer with an appearance of some dendrite-like branch structures outlined on its surface (Figure 13a). This explosive exothermic reaction with the detachment/ejection of the reacted system could take place serval times once the deposited multilayer reactants reach a critical thickness (Figure 13a’). The explosive reaction could also occur during the last step of depositing the last upper reactant layer, which lets only the iRMS motifs lodged into the litho-patterned photoresist trenches appear on the wafer surface. These embedded iRMS did not undergo ignition due to their direct contact with the substrate having slightly higher thermal conductivity compared to the one of the photoresist (Figure 13a). Images in Figure 13b,b’ present the patterned wafer surface morphology after the photoresist lift-off.

To overcome these encountered problems, which mostly are related to the high enthalpy of mixing/formation leading to an explosive reaction and the high inter-diffusion rate leading to spontaneous self-ignition, the modulation of the stored chemical energy and the rate of heat release would induce a substantial change in the characteristics of the reaction’s behavior. Accordingly, a reactivity tuning approach based on alternately stacking two different reactive multilayer systems, respectively, with different enthalpies of the mixing and formation of intermetallic compounds as well as different inter-diffusion rates will be investigated in the next section.

### 3.3. Pd/Al-Ni/Al Multi-Section (MS)-iRMS Grown on a SiO_2_-Coated Si-Wafer (MS-iRMS Expected Total Thickness ~3 μm)

**Figure 14:**
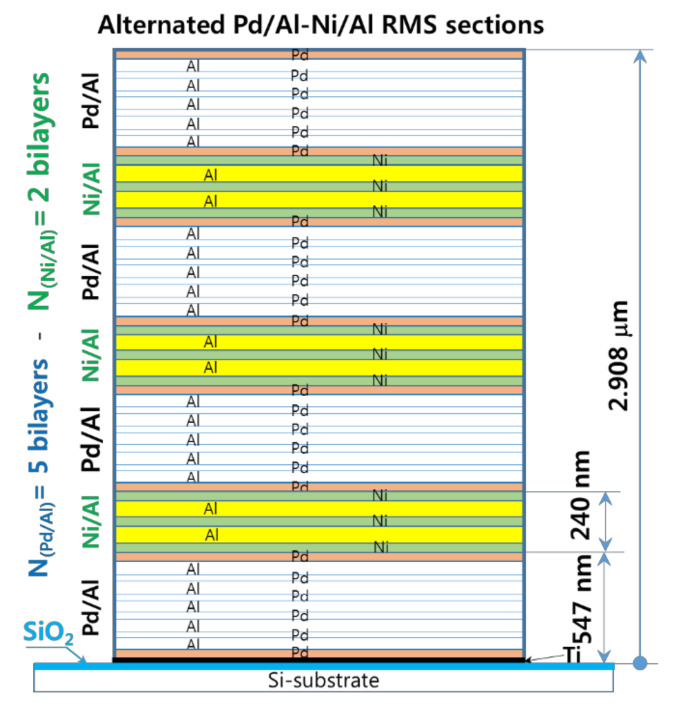
A cross-sectional schematic of the Pd/Al-Ni/Al MS-RMS design showing alternating sections of reactive materials with the corresponding section thicknesses. Design parameters: Pd(47 nm)/Al(53 nm) 5 bilayers, Ni(40 nm)/Al(60 nm) 2 bilayers, Pd/Al-section thickness S_hRMS_ = 500 nm (4 sections) and Ni/Al-section thickness S_mRMS_ = 200 nm (3 sections).

As the experiments outlined above demonstrate, for an RMS deposited on a substrate that has a low thermal conductivity, the high exothermic enthalpy of reactants mixing, and the resulting high temperature systematically lead either to spontaneous self-ignition during reactant deposition or to an explosive reaction with the detachment/ejection of the reacted product. Therefore, the modulation of the released heat and its subsequent temperature by stacking two reactive material systems with high and medium energy release (designed as hRMS and mRMS), respectively, is considered to be a potential solution. In this context, we investigated a sandwiched hRMS-mRMS reactive structure (see Figure 14), wherein the alternating hRMS sections correspond to the already used Pd/Al-RMS that had a higher enthalpy of mixing, and the alternating mRMS sections corresponded to the Ni/Al-RMS that had a medium enthalpy of mixing. The reactive Ni/Al system was selected for the relatively small difference between the CTE value of nickel (12 mm/mm.°C) and that of palladium (11.7 mm/mm.°C) in the reactive Pd/Al system. Indeed, as the temperature is increased, a CTE mismatch will induce compressive stress in a multilayer system that has a higher CTE value, and this tends to inhibit atomic diffusion into compressed system layers [37]. Hence, such effect would increase the activation energy of ignition, delay the intermixing process, and also reduce the propagation of the reaction rate [38]. For the dimensions of the sections in the reactive system structure, the thickness of the Pd/Al-hRMS section (S_hRMS_) was selected to be 0.5 μm to avoid spontaneous self-ignition, which was previously demonstrated to take place in Pd/Al-RMS on a SiO_2_-coated Si-substrate once the sputter deposition exceeded 1 μm. Moreover, since the CTE of Ni/Al-mRMS is slightly higher than that of Pd/Al-hRMS, the thickness of the Ni/Al-mRMS section (S_mRMS_) was selected to be smaller or equal to 0.2 μm, and, thus, its resulting volume would allow low tensile stress in the high exothermic Pd/Al system sections of the major volume fraction in the structure and would, consequently, avoid any noticeable change in the behavior of its properties. 

Furthermore, as Pd and Ni reactant components have a very negligible enthalpy of formation and mixing and could form a nearly ideal solution [39,40], therefore, to minimize further heat accumulation, which reduces the risk of spontaneous reactions and more premature interdiffusion during the deposition of hRMS-mRMS reactive sections, each Pd/Al-hRMS section deposition started and ended with the Pd layer and each Ni/Al-mRMS section deposition started and ended with the Ni layer. This structural arrangement ensures that Pd and Ni layers are always in contact at the interfaces between both hRMS and mRMS sections.

#### 3.3.1. Microstructural Characterization of the as-Deposited Pd/Al-Ni/Al MS-iRMS

**Figure 15:**
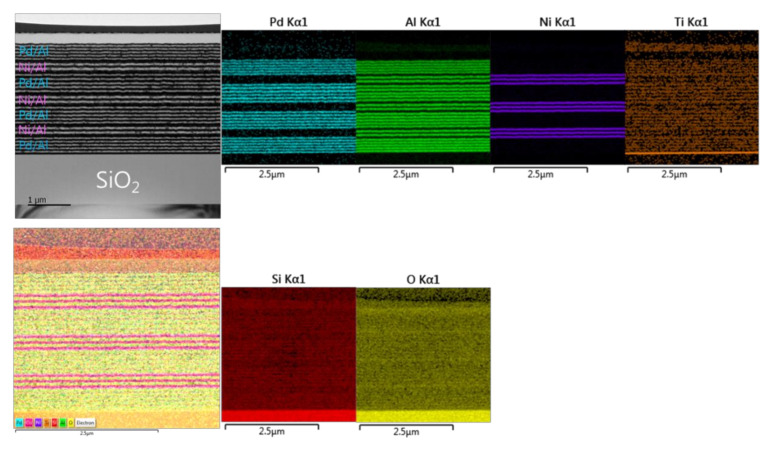
TEM cross section image (**upper left**) and the corresponding EDS elemental analysis (**upper right** and **lower left and right**) for the microstructural characterization of the as-deposited Pd/Al-Ni/Al MS-iRMS on the SiO_2_-coated Si-wafer.

Figure 15 presents TEM and EDS microstructural analyses of a Pd/Al-Ni/Al reactive multi-section system grown on SiO_2_-coated Si-wafer. The TEM cross section shows a total grown Pd/Al-Ni/Al system composed of four Pd/Al hRMS sections alternating with three Ni/Al mRMS sections. Both under-systems, hRMS and mRMS, have the same bilayer period δ of 100 nm and the same molar ratio of 1:1. The TEM and EDS images show a neat typical multilayered structure where the alternating single layers are clearly identified in each section composing the whole reactive multi-section system. The clear interfaces between the reactant layers in each section reveal that neither pre-intermixing reaction nor spontaneous self-ignition and reaction propagation occurred during the sputtering process. The thicknesses of the single deposited layers of each section approach the expected nominal values corresponding to a 1:1 atomic ratio: 47 and 53 nm for the bilayer Pd/Al and 40 and 60 nm for the bilayer Ni/Al.

**Figure 16:**
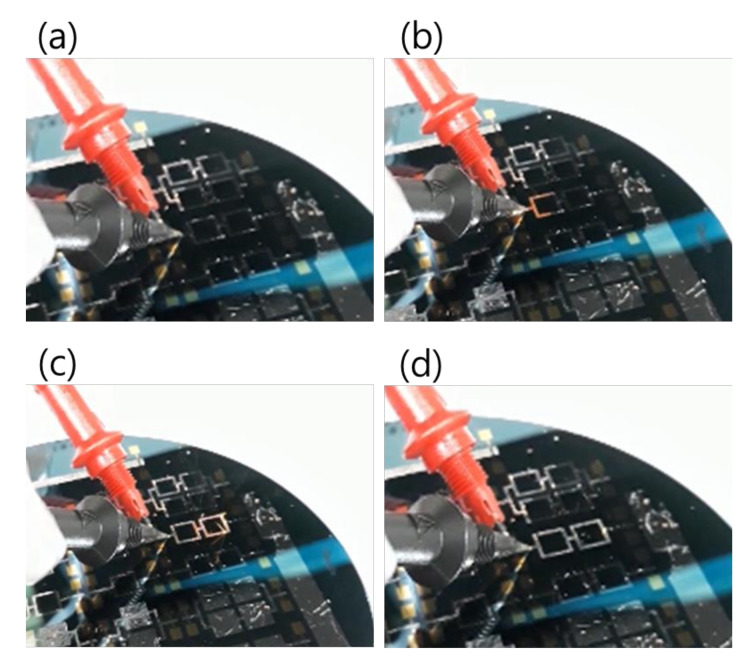
Images showing reaction front propagation steps across a patterned Pd/Al-Ni/Al MS-iRMS of squared frames grown on a SiO_2_/Si-wafer. (**a**) Initiation step; (**b**,**c**) Self-maintained propagating reaction steps; (**d**) Reaction product morphology after the reaction step.

**Figure 17:**
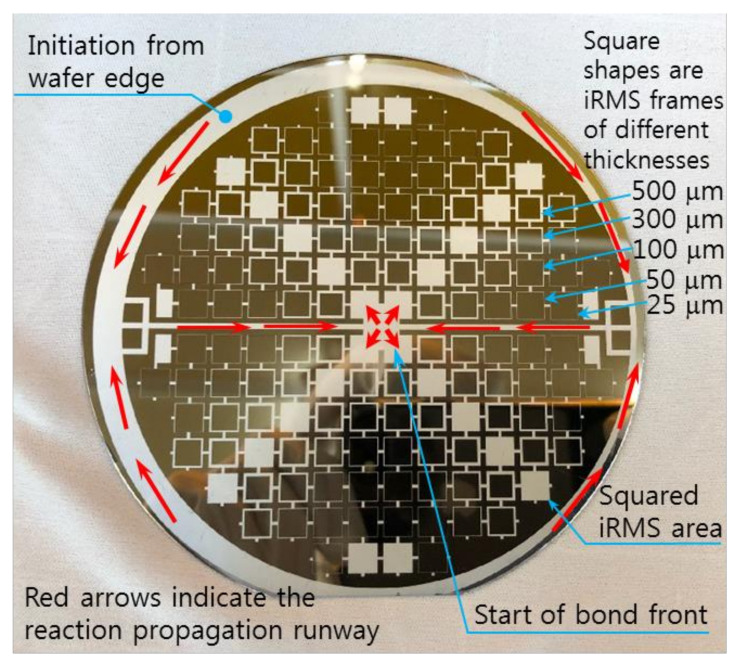
Example of an interconnected patterning design allowing the propagating reaction to run through the entire iRMS on the wafer.

Regarding the electrical ignition of this reactive multi-section structure, it was found that it could easily be initiated at room temperature by applying an electric DC pulse of 10 V/max. 1 A. After initiation, the reaction front spread evenly through the structured Pd/Al-Ni/Al MS-iRMS in a self-sustained manner without any further energy supply (no preheating). The images in Figure 16 show the expansion progress of a bright glow representing the steady self-propagation of the reaction front along Pd/Al-Ni/Al MS-iRMS patterned in the linked square frame structure. The patterned Pd/Al-Ni/Al MS-iRMS frame width presented here was 500 μm, and it was also demonstrated that the minimum bond frame width showing a self-propagating exothermic reaction could be reduced to 25 μm. For the self-propagating reaction front to pass through complex frame geometries, the iRMS patterning frames must be connected to each other to assure the reaction propagation on the whole patterned wafer. Figure 17 shows an example of interconnected patterning design allowing the propagation reaction to run through the entire iRMS on the wafer. In this example, the ignition could be initiated at the edge of the wafer with the resulting reaction guided to the wafer’s center from which it propagates to all wafer regions. Furthermore, it should be noted that the ignited reaction in Pd/Al-Ni/Al MS-iRMS propagated in an explosive manner, causing the ejection of small debris particles from the reacted product. However, the main reacted multi-section structure showed good adhesion to the substrate.

#### 3.3.2. Thermal Characterization of the as-Deposited Pd/Al-Ni/Al MS-iRMS

**Figure 18:**
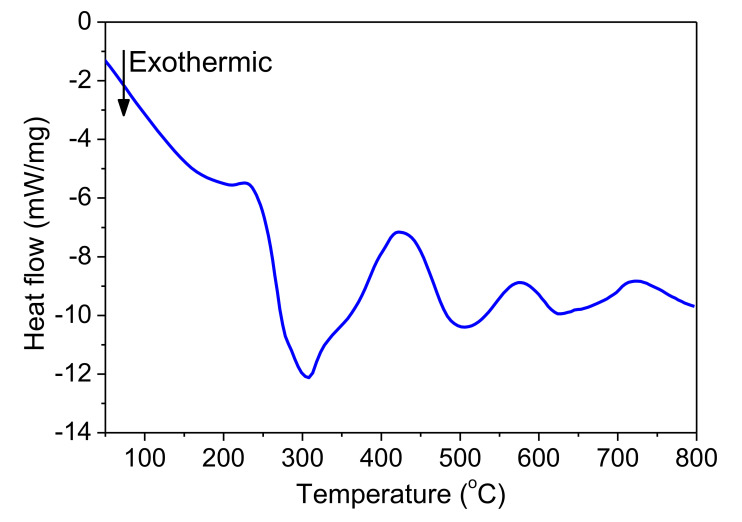
DSC characterization of the as-deposited Pd/Al-Ni/Al MS-iRMS.

Figure 18 shows the DSC curve of the Pd/Al-Ni/Al MS-RMS freestanding nano-foil grown on the SiO_2_/Si-wafer coated with a Cu layer of 250 nm and released by immersion in an acidic solution bath. Three peaks that spread out towards high temperatures are observed. Such behavior is similar to the case of the usual DSC trace measured on a single Pd/Al RMS, in which the activation energy of the diffusion of Pd in Al is on the order of 190.6 kJ/mol [41], but in contrast with the case for a Ni/Al reactive system. For this, the exothermic peaks typically appear at lower temperatures in a range from 240 °C to 400 °C [1,12,16,18,42], since the activation energy of the diffusion of Ni in Al is slightly lower on the order of 145.8 kJ/mol [41]. Thence, the measured exothermic reaction of the Pd/Al-Ni/Al MS-RMS would be a superposition of the exothermic peaks of Pd/Al sections with those of the Ni/Al sections. The shift of the Ni/Al exothermic peaks to higher temperatures to superpose with those of the Pd/Al system could be related to the expansion difference of the two alternating Pd/Al and Ni/Al reactive systems (Ni_(CTE)_ > Pd_(CTE)_). Thus, internal compressive stress is induced into the Ni/Al sections. Such a stress state reduces the Ni diffusion rate in the Al and consequently increases the Ni diffusion activation energy [37,38], which constrains the Ni mobility such that it takes place at a higher temperature for the reaction. The extracted total exothermic heat released from the reactive Pd/Al-Ni/Al system was determined from the DSC measurement to be equal to 1191.7 J/g, an amount that is slightly higher than the theoretically predicted enthalpy of formation for the AlNi reaction product (ΔH_AlNi_ = 1120 J/g) [36] and slightly lower than that for the AlPd reaction product (ΔH_AlPd_ = 1260 J/g) [36]. Hence, the energy produced in the Pd/Al-Ni/Al system confirms that the stacking of combined hRMS/mRMS reactive systems, with different reaction enthalpy, is an efficient tool for heat tuning (either heat storing or heat releasing; both must be addressed in reactive bonding applications).

#### 3.3.3. Microstructural Characterization of the Reacted Pd/Al-Ni/Al MS-iRMS

**Figure 19:**
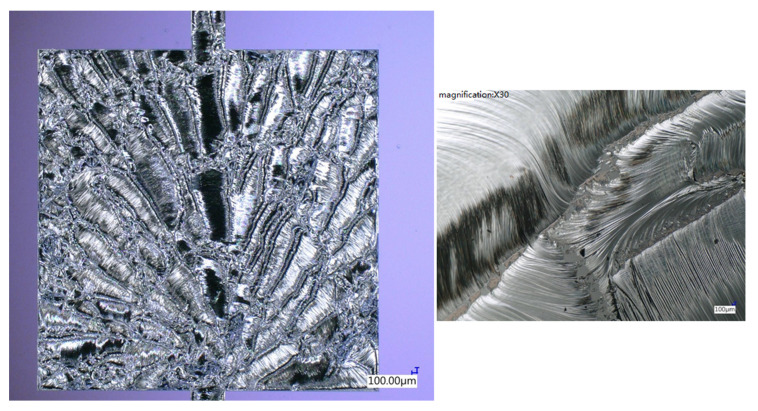
Optical microscopic image showing the microstructural morphology of the reacted Pd/Al-Ni/Al MS-iRMS. The right image is a magnified view depicting inter-dendritic regions formed during reaction propagation.

Figure 19 shows an optical microscopic image of the top-view surface morphology of the reacted Pd/Al-Ni/Al MS-iRMS. The reaction was ignited with an electrical DC pulse (10 V/max. 1 A) at room temperature, which resulted in a self-maintained propagating reaction that spread over the whole patterned RMS area within a few milliseconds. The reaction product surface morphology exhibited a spherulitic-like dendrite crystallite structure [43]. The dendrite orientations were seen to have emerged from an origin and extended toward the reaction front propagation direction to form maple leaf-like patterns. The manifestation of the reaction product in dendritic form is a common solid crystallization grown from the melt under a fast-cooling rate [44,45]. The steeper temperature gradients, systemically established behind the RMS reaction front propagation, would spontaneously introduce significant thermal stress gradients associated with the solidification process [46]. Further, thermal stress states could also increase more if the thermal expansion coefficients of the dual section materials are significantly different [38]. These factors all generated stresses that enhanced the reacted product’s susceptibility to cracking or disintegration through the associated strains. Note that the self-sustained propagating reaction in our electrical ignited sample occurred in an explosive manner, ejecting reacted product in debris particles detached from the coarsened dendrite trunks and, hence, inter-dendritic regions were formed, as seen in the magnified view in Figure 19 (right). From this exhibited behavior, it is evident that, in the multi-section stack of the iRMS, adopting a section type composed of reactive reactants that have a high reaction product ductility property at room temperature would attenuate the propagating reaction effect, causing disintegrated product.

**Figure 20:**
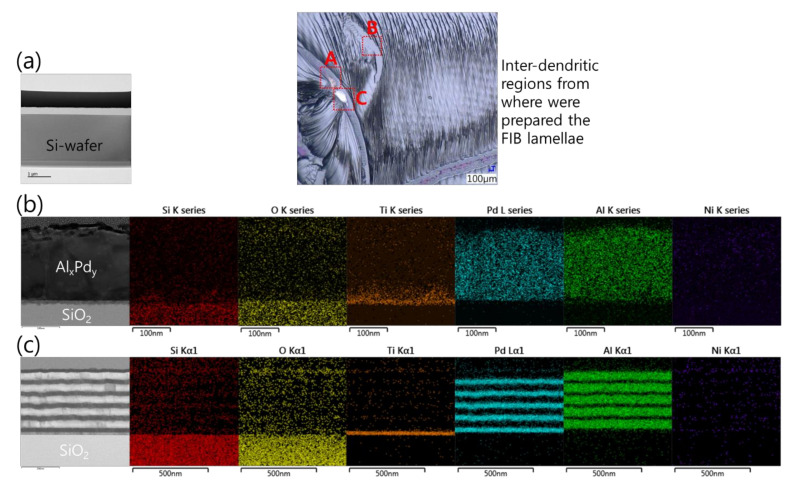
TEM cross section images and EDS analyses performed on different FIB cross sections selected from different regions of the upper surface of the reacted Pd/Al-Ni/Al MS-iRMS (see the inset optical microscopic image). (**a**) FIB cross section from a bare inter-dendritic area (zone A); (**b**) FIB cross section from a region where a partial reacted product was detached (zone B); (**c**) FIB cross section from a region where a partial unreacted multilayer stack residue was still attached to the substrate (zone C).

Sample preparation by FIB for a cross-sectional analysis of the Pd/Al-Ni/Al MS-iRMS reaction product was not possible. The intense induced thermal residual stresses in the grown dendrite trunks made them very friable and fragile and, thus, the prepared lamella structure easily collapsed by the ion beam milling. Notwithstanding this obstacle, some cross-sectional analyses were carried out in order to gain more insights into the effects controlling the reaction propagation and the reaction product formation. The inset optical microscopic image in Figure 20 indicates different area zones where FIB-prepared cross section specimens were cut from a sample of the Pd/Al-Ni/Al MS-iRMS reacted product. Figure 20a depicts a TEM image of an FIB cross section of a bare area located between the inter-dendritic regions (zone A). The image confirmed that the ejected reaction product was accompanied by the local fragment detachment of the SiO_2_ thermal isolating layer. Figure 20b (left) depicts a TEM image of a FIB cross section undertaken in a region where a partial reacted product was detached/ejected during the reaction propagation (zone B). The remaining product was found to belong to the first deposited Pd/Al section on the Ti/SiO_2_/Si-substrate. This has been confirmed by an EDS analysis, which revealed only the formation of Al_x_Pd_y_ intermetallic compound (traces of Pd and Al elements) and the absence of nickel, which should structurally be in the subsequent Ni/Al section if it were present (Figure 20b, right). Figure 20c (left) depicts a TEM image of a FIB cross section of a region from where a partially reacted product was detached/ejected (zone C); the image shows that it concerns a remaining underneath part that corresponds to unreacted Pd/Al multilayer stack residue of the first section in direct contact with the Ti/SiO_2_/Si-substrate. The elemental chemical composition of the intact Pd/Al layers in this unreacted residue was confirmed by the EDS analysis in Figure 20c (right).

**Figure 21:**
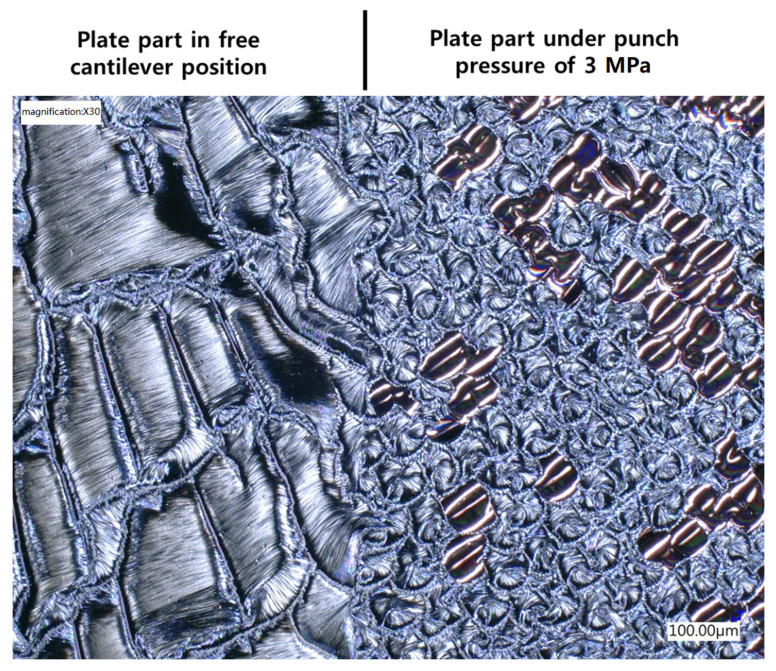
Optical microscope image showing the effect of the ignition under pressure on the reaction product microstructure configuration for Pd/Al-Ni/Al MS-iRMS.

One more interesting point to raise is that it was found that the dendritic structure of the reaction product took place only when the iRMS was ignited in an open-air atmosphere or vacuum chamber. However, when the iRMS was ignited under a high punch pressure, the reaction product was a dense nodular-like structure. Figure 21 shows an optical microscopic image of the reaction product microstructure resulting from a multi-section Pd/Al-Ni/Al iRMS for which the substrate plate was set, at the ignition process step, in a press machine that was as follows: One part of the substrate plate was in a free cantilever position, and the second remaining part was covered by a SiO_2_/Si-plate. Together, these were interposed between the press flat punches where they were uniaxially compressed under a pressure of 3 MPa. After the ignition, the propagation of the reaction extended, both, over the entire un-pressed MS-RMS free part and through the entire pressed MS-RMS part sandwiched between the substrate plates. The results demonstrate that pressure, which is commonly used in reactive bonding, can prevent the detachment/ejection of the reaction product in bonding processes. 

**Figure 22:**
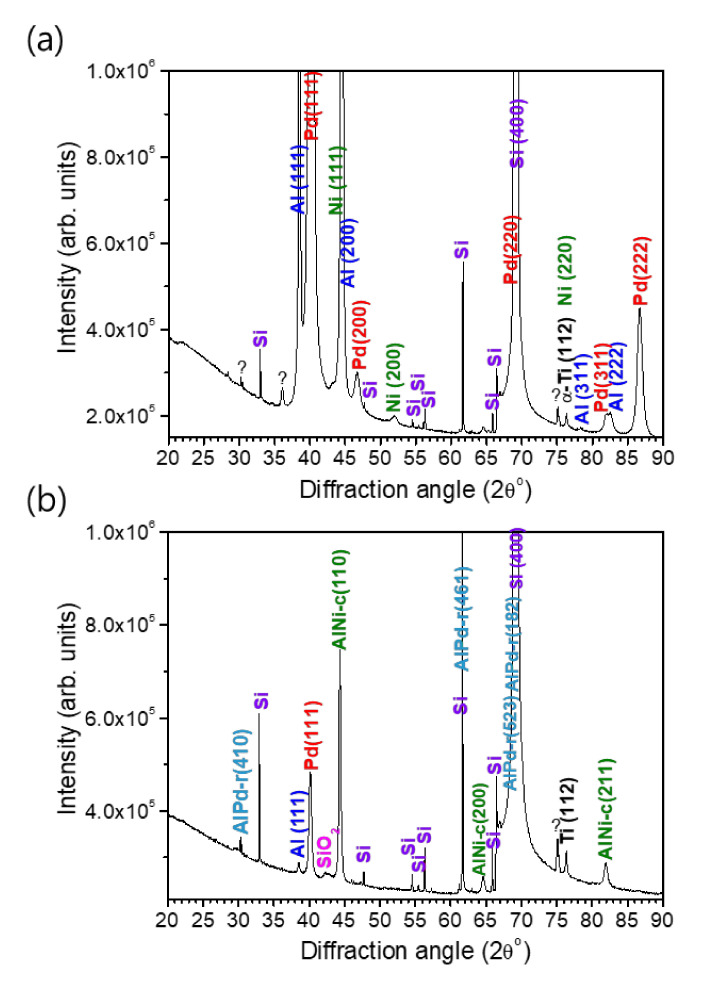
XRD patterns of Pd/Al-Ni/Al MS-iRMS on the SiO_2_/Si-substrate. (**a**) As-deposited sample; (**b**) Reaction product after electrical ignition.

Since a full intact cross-sectional lamella of the reaction product of the multi-section Pd/Al-Ni/Al nano-reactive multilayer system could not be obtained to perform structural and compositional analyses on, a complementary analysis by XRD was carried out on both the as-deposited and reacted Pd/Al-Ni/Al MS-iRMS. Thus, the crystalline structure of the multilayer materials and the phase composition of reacted multi-section materials were examined. The XRD patterns of as-deposited and reacted Pd/Al-Ni/Al MS-iRMS on the SiO_2_/Si-substrate are shown in Figure 22.

For the as-deposited Pd/Al-Ni/Al MS-iRMS (Figure 22a), the diffraction peaks indexed to Al(111), Al(200), Al(311), Al(222), Pd(111), Pd(200), Pd(220), Pd(311), Pd(222), Ni(111), Ni (200), and Ni(220) indicate that the Al, Pd, and Ni elements forming the Pd/Al and Ni/Al multilayers in iRMS sections are all present in the crystalline phase (the used ICDD cards for peaks indexing are: #04-0787 for Al, #00-046-1043 for Pd, and #00-004-0850 for Ni). For the Pd/Al-Ni/Al MS-iRMS reaction product (Figure 22b), the new groups of major diffraction peaks indexed to AlPd-r(410), AlPd-r(461), AlPd-r(523), AlPd-r(182) and AlNi-c(110), AlNi-c(200), and AlNi-c(211) (r and c, respectively, denote rhombohedral and cubic) indicate that AlPd and AlNi are the major resultant intermetallic compounds of the final products of the exothermic reaction originating, respectively, from the Pd/Al and Ni/Al multilayer films. The indexed peaks were found to match the rhombohedral crystalline structure for the AlPd reaction product and cubic crystalline structure for the AlNi reaction product (corresponding ICDD cards are #00-031-0027 for r-AlPd and #01-073-2594 for c-AlNi). It should also be mentioned that some additional diffraction peaks were observed in both XRD patterns, before and after the exothermic reaction. These peaks essentially originated from the SO_2_/Si-substrate and the Ti adhesive layer. The XRD data of the reaction product also revealed unreacted Pd/Al multilayer stack residues that were still attached to the substrate after the reaction propagation. They were manifested by a diffraction continuity of Pd (111) and Al (111) peaks, but with a lower intensity magnitude, indicating the existence of only a small, dispersed amount of such unreacted residue resulting from the incomplete reaction of the first deposited Pd/Al section. Furthermore, the unknown observed peaks in the XRD data of the as-deposited multi-section iRMS could be related to a solid solution reaction occurring from the pre-intermixing at the multilayer interfaces during the deposition process. Consequently, when a multi-section iRMS, with alternating sections of Pd/Al and Ni/Al multilayers undergoes an exothermic reaction process, the intermetallic AlPd and AlNi compounds are found to be the dominantly formed products.

To briefly summarize the main findings of this investigation, the combined hRMS/mRMS reactive systems demonstrated two important beneficial impacts on the integrated multi-section reactive system: first, avoiding the spontaneous self-ignition reaction during the deposition process; and second, assuring iRMS ignition with a sustained reaction propagation simply by electrical triggering at room temperature.

## 4. Conclusions

We proved experimentally that, for the integration of a high energetic RMS on Si-wafer substrates for reactive bonding, two main conflicting issues should be overcome: (i) the heat accumulation and consequent spontaneous self-ignition reaction and (ii) the heat loss through the sink substrate and consequent reaction propagation quenching.

In this work, we investigated the integration of a highly energetic Pd/Al nano-reactive multilayer system on silicon wafers. To address the heat control difficulties in and around the integrated reactive system, Pd/Al-iRMS was deposited on different wafers of different thermal conductivities: a bare Si-wafer, RuO_x_ or PdO_x_ layers buffering Si-wafers, and a SiO_2_-coated Si-wafer. The growth of Pd/Al-iRMS on a bare silicon wafer of higher thermal conductivity could reach a total thickness over 1 μm without the upsurging of a spontaneous self-ignition reaction during the deposition process. Nonetheless, for this structure, due to the efficient heat sink by the intimate thermal contact of RMS with the Si-wafer substrate, in order to be electrically ignitable at RT with a stable reaction front propagation, the effective Pd/Al-iRMS thickness must start from 5 μm and over, which is technically not desirable (due to high internal stress generation in the iRMS and lift-off pattering limitations). However, for the other coated Si-wafers, those with an instant thermal barrier made of metal oxide RuO_x_ or PdO_x_ buffers causing moderate thermal conductivity and the other with a SiO_2_ layer of lower thermal conductivity, the Pd/Al-iRMS growth on all of them, specifically with a bilayer thickness of 100 nm or less, underwent systematic spontaneous self-ignition surging during the deposition process upon reaching a thickness of around 1 μm.

To overcome these encountered issues, we investigated a solution based on the tuning of the reaction heat release by combining RMS of a different exothermic reaction enthalpy. This type of heat modulation has direct control over the desired generation of the local temperature and reaction propagation characteristics. The materialization of this solution was accomplished by alternately stacking sections of metallic reactive multilayer Pd/Al and Ni/Al systems that, respectively, have a high and medium exothermic enthalpy of reaction. The grown heterostructure was successfully deposited directly on a SiO_2_-coated Si-wafer with a bilayer of 100 nm and a total thickness of 3 μm without any spontaneous upsurge of self-ignition. Moreover, the ignition of the alternating multi-section Pd/Al-Ni/Al iRMS structure was successfully initiated with a DC pulse of 10 V/max. 1 A at RT ambient without requiring any external preheating supplement, and this was possible for the whole patterned Pd/Al-Ni/Al MS-iRMS units on the wafer. Furthermore, the reaction ignition in this heterogeneous multilayer, multi-section structure showed a stable self-sustained propagating reaction front along the whole patterned Pd/Al-Ni/Al MS-iRMS path without any local propagating reaction quenching. 

The DSC analyses confirmed the predicted heat output modulation in the combined Pd/Al-Ni/Al MS-iRMS structure compared to the single structured Pd/Al iRMS. The specific heat output by the combined reactive systems effectively resulted in a heat amount (1191.7 J/g) that was relatively lower than the theoretical exothermic heat (1260 J/g) related to a pure single reactive Pd/Al system. DSC results also revealed that the phase transformation mechanisms to promote the formation of intermetallic compounds took place simultaneously, in both Pd/Al- and Ni/Al-stacked RMS, at the same temperatures and along the same temperature range. This thermal uniformity is important during the reaction of different stacked reactive systems; it avoids the reaction decoupling effect on the heat wave and the reaction propagations in each section of the stacked systems.

The analysis by X-ray diffraction of the Pd/Al-Ni/Al MS-iRMS reacted structure confirmed the formation of intermetallic compounds: mainly an AlPd reaction product with a rhombohedral crystalline structure, formed through the Pd/Al stack system, and an AlNi reaction product with a cubic crystalline structure, formed through the PNi/Al stack system. Both structures were mostly dominant in the formed reaction products. 

This approach of alternating reactive sections with interfaces of different reactive affinity and different output energy demonstrated an efficient potential for the integration of the reactive system. It allowed the limitation of heat accumulation during the film growth and the tuning of heat source without changing the iRMS structural architecture. However, further investigation regarding the design parameters of the Pd/Al-Ni/Al MS-iRMS is still needed to optimize the reactive system integration to supply enough output energy capable of metal solder melting as well as compensating the heat conduction into the bonding partners. Also, given that the intermetallic compounds are highly brittle, to preserve the integrity and mechanical reliability of the reaction product’s material after the reaction propagation through the iRMS, research on improving the ductility of the reaction product at room temperature is necessary. The ductility dampens the residual internal stress in the reacted iRMS product and, thus, avoids the explosive reaction with particle ejection during the formation of the reaction product. Finally, we can conclude that the proposed solution based on stacking alternating reactive systems with different exothermic heat of reactions provides an efficient potential method that could overcome the different issues of integrated RMS for bonding applications in microelectronics and microsystems technology.

## Figures and Tables

**Table 1:** Thermal conductivity values of the covering layers used in the preparation of Si-wafers before the integrated reactive multilayer systems (iRMS) deposition.

Wafer and Covering Layers	Wafer and Covering Layer Thicknesses	Thermal Conductivity (W/m.K)	Equivalent Thermal Conductivity (W/m.K)
Silicon (wafer)	500 μm (bare wafer)	148 [27]	
SiO_2_	1 µm	1.4 [27]	
SiN_x_/RuO_x_/Cr	50 nm/60 nm/10 nm	25/50/91.3 [28,29,30]	36.2
SiN_x_/PdO_x_/Cr	50 nm/60 nm/10 nm	25/37.5 ^★^/91.3	32.3
Photoresist	3 μm	0.19 [31]	

^★^ As the PdO_x_ thermal conductivity value has not been measured and was also not available in the literature, and since the palladium oxidation at low temperature is basically not complete, we estimated its thermal conductivity to be half of the pure metallic palladium 75 W/m.K [30].
